# Supplementary material for: Origin and arrangement of actin filaments for gliding motility in apicomplexan parasites revealed by cryo-electron tomography
Source: Nat Commun. 2023 Aug 9;14:4800. doi: 10.1038/s41467-023-40520-6 (PMC10412601; doi:10.1038/s41467-023-40520-6)
Supplement: Supplementary file 1 — Supplementary Information [file 41467_2023_40520_MOESM1_ESM.pdf]

Supplementary Table 1. Cryo-ET data collection and processing parameters.

	Cryo-ET Data Collection and Processing		
	Wildtype *C. parvum*	Wildtype *T. gondii*	FRM1-iKD *T. gondii*
Magnification	33,000x	33,000x	33,000x
Voltage (keV)	300	300	300
Electron exposure (e^-/Å^2)	140	140	140
Defocus range (µm)	-2 to -4	-2 to -4	-2 to -4
Pixel size (Å)	2.65	2.65	2.65
Tilt range (°)	-60 to 60	-60 to 60	-60 to 60
Frames per tilt	4	4	4
Exposure time per frame (s)	0.1	0.1	0.1
Volta Phase Plate	Yes	Yes	Yes
Detector	K3	K3	K3
Energy filter width (eV)	20	20	20
Spherical aberration (mm)	2.7	2.7	2.7
Tomogram reconstruction software	IMOD	IMOD	IMOD
Tilt series alignment method	10 nm gold fiducials	10 nm gold fiducials	10 nm gold fiducials
CTF correction	No	No	No
Tomogram reconstruction algorithm	Weighted back projection with SIRT-like filter	Weighted back projection with SIRT-like filter	Weighted back projection with SIRT-like filter

Supplementary Table 2. Subtomogram averaging parameters for *C. parvum* preconoidal rings.

	Subtomogram Averaging Parameters		
	C. parvum preconoidal rings	*C. parvum* upper preconoidal ring	*C. parvum* lower preconoidal ring
Software used	Dynamo	Dynamo	Dynamo
Mask shape	Ellipsoid	Ellipsoid	Ellipsoid
**Numerical parameters**			
Iterations	3	3	3
References	1	1	1
Cone aperture (°)	30	12	12
Cone sampling (°)	7.5	2	2
Azymuth rotation range (°)	30	12	12
Azymuth rotation sampling (°)	7.5	2	2
Refine	5	4	4
Refine factor	2	2	2
High pass	2	2	2
Low pass	48	40	40
Symmetry	C1	C1	C1
Particle dimensions (pixels)	90 (downsampled by 4)	50 (downsampled by 4)	50 (downsampled by 4)
Shift limits (pixels)	10 10 10	10 10 10	10 10 10
Shift limiting way	1	2	2
Threshold parameter	0.67	0.67	0.67
Threshold modus	5	5	5

Supplementary Table 3. Subtomogram averaging parameters for wildtype *T. gondii* preconoidal rings.

	Subtomogram Averaging Parameters		
	T. gondii preconoidal rings	*T. gondii* upper preconoidal ring	*T. gondii* lower preconoidal ring
Software used	Dynamo	Dynamo	Dynamo
Mask shape	Ellipsoid	Ellipsoid	Ellipsoid
**Numerical parameters**			
Iterations	3	3	3
References	1	1	1
Cone aperture (°)	30	12	12
Cone sampling (°)	7.5	2	2
Azymuth rotation range (°)	30	12	12
Azymuth rotation sampling (°)	7.5	2	2
Refine	5	4	4
Refine factor	2	2	2
High pass	2	2	2
Low pass	48	40	40
Symmetry	C1	C1	C1
Particle dimensions (pixels)	90	50 (downsampled by 4)	50 (downsampled by 4)
Shift limits (pixels)	10 10 10	10 10 10	10 10 10
Shift limiting way	1	2	2
Threshold parameter	0.67	0.67	0.67
Threshold modus	5	5	5

Supplementary Table 4. Subtomogram averaging parameters for FRM1-iKD *T. gondii* preconoidal rings.

	**Subtomogram Averaging Parameters**		
	FRM1-iKD *T. gondii* preconoidal rings	FRM1-iKD *T. gondii* upper preconoidal ring	FRM1-iKD *T. gondii* lower preconoidal ring
Software used	Dynamo	Dynamo	Dynamo
Mask shape	Ellipsoid	Ellipsoid	Ellipsoid
**Numerical parameters**			
Iterations	3	3	3
References	1	1	1
Cone aperture (°)	30	12	12
Cone sampling (°)	7.5	2	2
Azymuth rotation range (°)	30	12	12
Azymuth rotation sampling (°)	7.5	2	2
Refine	5	4	4
Refine factor	2	2	2
High pass	2	2	2
Low pass	48	40	40
Symmetry	C1	C1	C1
Particle dimensions (pixels)	90	50 (downsampled by 4)	50 (downsampled by 4)
Shift limits (pixels)	10 10 10	10 10 10	10 10 10
Shift limiting way	1	2	2
Threshold parameter	0.67	0.67	0.67
Threshold modus	5	5	5

Supplementary Table 5. Subtomogram averaging parameters for *C. parvum* IMC surface filament.

	**Subtomogram Averaging Parameters**	
	C. parvum IMC surface filament (per tomogram average)	*C. parvum* IMC surface filament (multireference alignment)
Software used	Dynamo	Dynamo
Mask shape	Cylinder	Cylinder
**Numerical parameters**		
Iterations	3	3
References	1	3
Cone aperture (°)	24	24
Cone sampling (°)	4	4
Azymuth rotation range (°)	60	60
Azymuth rotation sampling (°)	10	10
Refine	6	6
Refine factor	2	2
High pass	2	2
Low pass	76	76
Symmetry	C1	C1
Particle dimensions (pixels)	40 (downsampled by 4)	40 (downsampled by 4)
Shift limits (pixels)	8 8 8	8 8 8
Shift limiting way	1	2
Threshold parameter	0.7	0.7
Threshold modus	5	5

Supplementary Table 6. Subtomogram averaging parameters for *C. parvum* basal IMC pore.

	**Subtomogram Averaging Parameters**	
	C. parvum basal IMC pore (unsymmetrized)	*C. parvum* basal IMC pore (symmetrized)
Software used	Dynamo	Dynamo
Mask shape	Ellipsoid	Ellipsoid
**Numerical parameters**		
Iterations	3	3
References	1	1
Cone aperture (°)	12	12
Cone sampling (°)	2	2
Azymuth rotation range (°)	45	12
Azymuth rotation sampling (°)	11.25	2
Refine	6	3
Refine factor	2	2
High pass	2	2
Low pass	170	170
Symmetry	C1	C8
Particle dimensions (pixels)	80 (downsampled by 4)	80 (downsampled by 4)
Shift limits (pixels)	20 20 20	4 4 4
Shift limiting way	1	2
Threshold parameter	0.7	1
Threshold modus	5	5

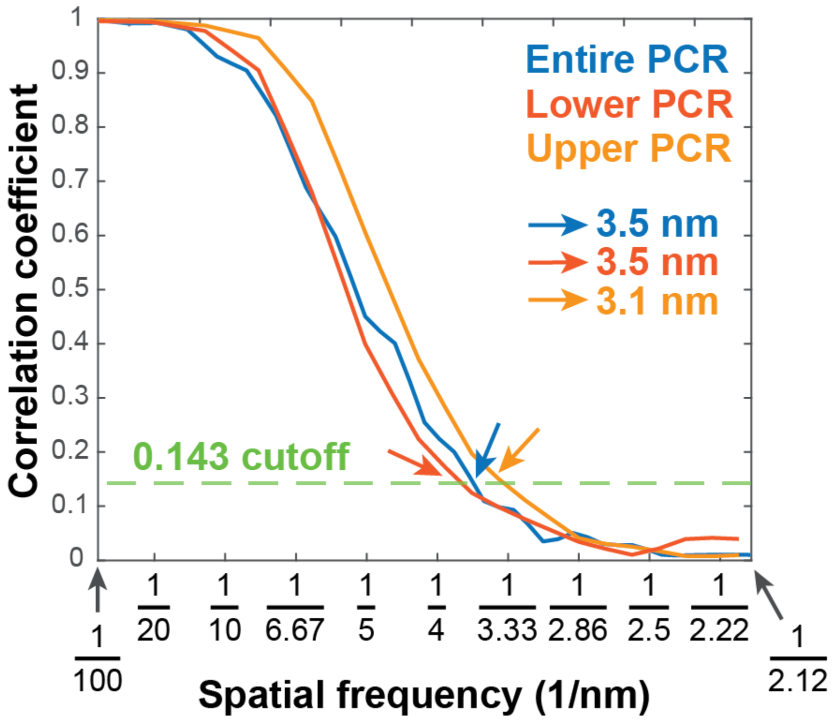

Supplementary Figure 1. Fourier shell correlation plot for the subtomogram averages of the *C. parvum* PCRs. The plot shows the FSC curves for the subtomogram averages of the entire PCR (blue), the upper PCR (orange) and the lower PCR (red) along with their resolution according to the 0.143 cutoff.

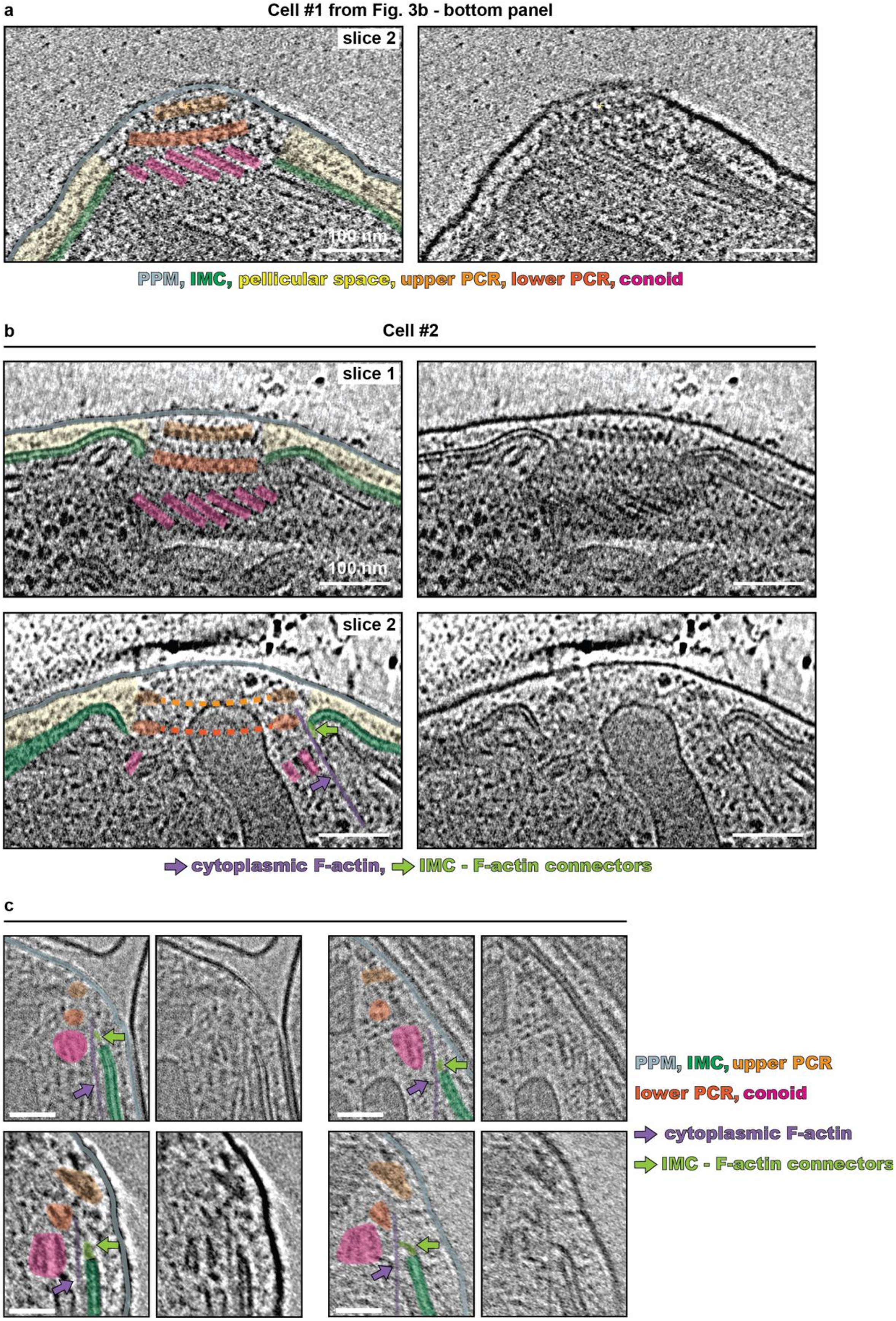
a
Cell #1 from Fig. 3b - bottom panel
slice 2
100 nm
PPM, IMC, pellicular space, upper PCR, lower PCR, conoid
b
Cell #2
slice 1
100 nm
slice 2
cytoplasmic F-actin, IMC - F-actin connectors
c
PPM, IMC, upper PCR
lower PCR, conoid
cytoplasmic F-actin
IMC - F-actin connectors

Supplementary Figure 2. **Retracted conoids in *C. parvum* sporozoites and the associated cytoplasmic F-actin**. **(a)** An additional 2-D slice of the *C. parvum* tomogram shown in Fig. 3b – lower panel – to unambiguously show the retracted conoid. **(b)** 2-D slices from another *C. parvum* sporozoite tomogram with a retracted conoid displaying F-actin gated into the cytoplasmic space and its interactions with the IMC collar via putative connector densities. **(c)** A gallery of 2-D slices from *C. parvum* sporozoite tomograms showing putative connector densities between cytoplasmic F-actin and IMC collar. Scale bars are 100 nm in panels a and b, and 50 nm in panel c.

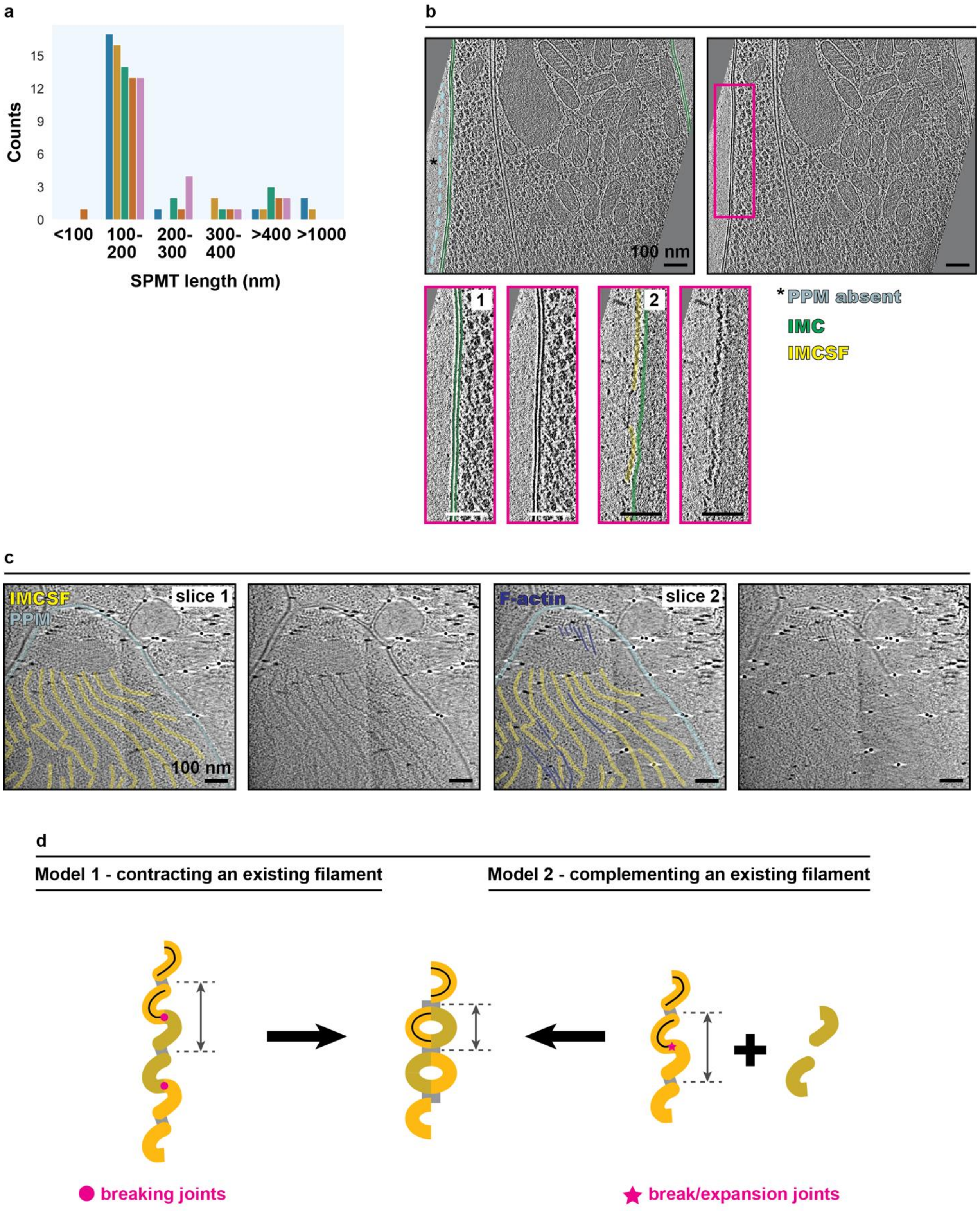

Supplementary Figure 3. Subpellicular microtubule and inner membrane complex surface filament architecture in *C. parvum* sporozoites (a) Histogram showing the distribution of SPMT lengths from 5 *C. parvum* cells with each color representing an

individual cell. The majority of SPMTs are shorter than 200 nm in length. **(b)** 2-D slices from a *C. parvum* tomogram with a lost or ruptured PPM (likely due to sample preparation). The pink box denotes the region enlarged in the panels below. The zoomed-in views show two different slices (numbered 1 and 2) with (left) and without (right) color overlay. The IMCSFs can be seen associated with the IMC even in the absence of the PPM. **(c)** 2-D slices from a tomogram with (left) and without (right) color overlays, showing disorganized IMCSFs further into the cell body (slice 1) and F-actin crossing over into adjacent inter-IMCSF space (slice 2). **(d)** Two hypothetical models for filament transition from the sawtooth conformation to the inter-connected loop conformation based on the structural details revealed by subtomogram averaging and the evidence for such transitions within the same filament *in situ*. Filament transition in both models involves a rearrangement of the S-shaped subunit of the sawtooth conformation. However, in model 1, it is achieved by contracting an existing filament (involving intrasubunit breakage), while in model 2, it is achieved by complementing an existing filament with additional components, accompanied by some minor rearrangements (again involving intrasubunit breaking or expansion). Scale bars are 100 nm.

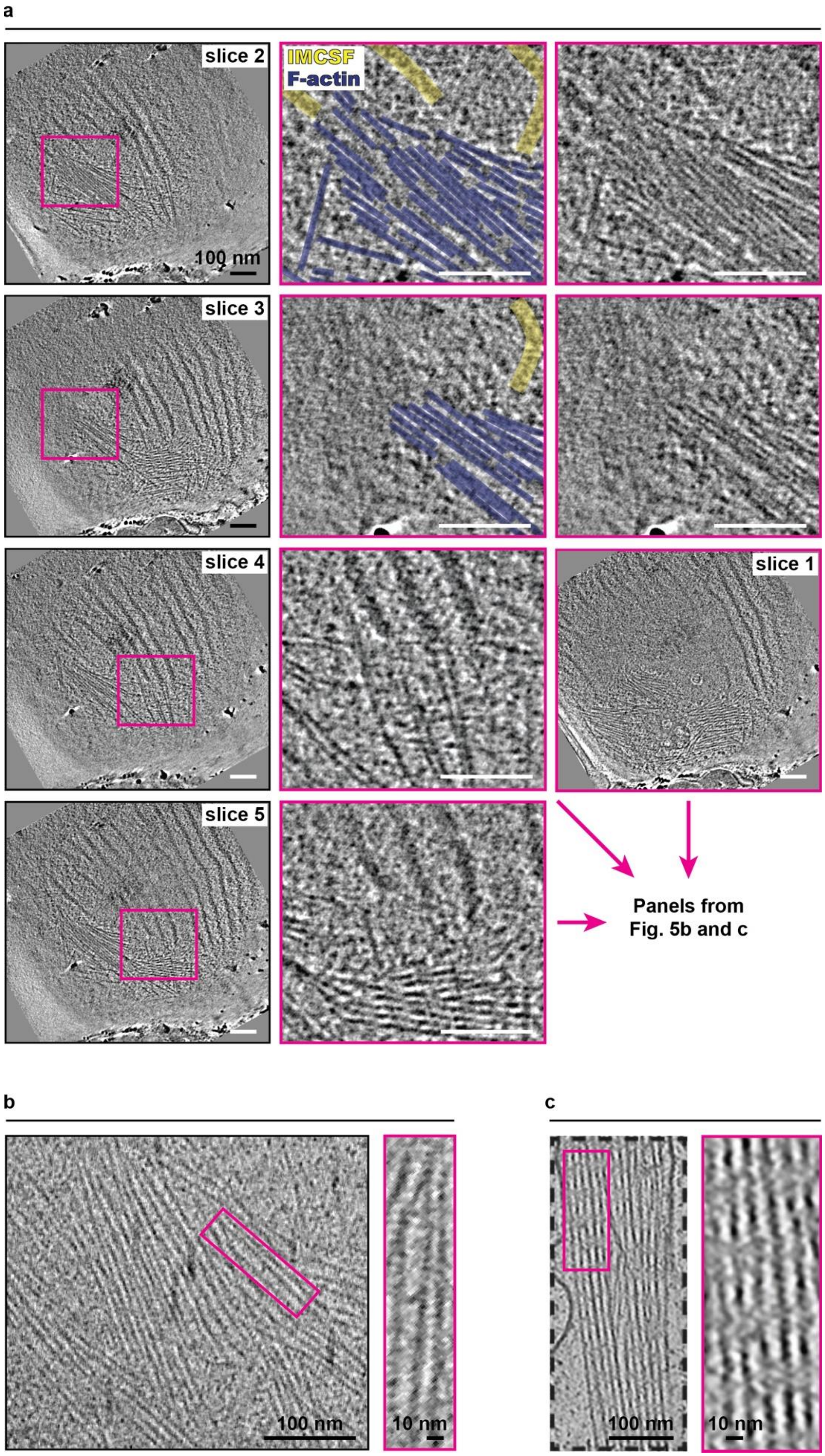

a
slice 2
IMCSF
F-actin
100 nm
slice 3
slice 4
slice 1
slice 5
Panels from
Fig. 5b and c
b
100 nm
10 nm
c
100 nm
10 nm

Supplementary Figure 4. F-actin organization at the basal end of *C. parvum* sporozoites. (a) Numerous slices through the basal end tomogram shown in Fig. 5b with the numbering of the slices consistent between the two figures. These panels show additional slices through the tomogram with and without color overlays. Pink boxes denote regions that are enlarged. The enlarged panels corresponding to slices 2 and 3 include color overlays while the color overlays for those corresponding to slices 4 and 5 are shown in Fig. 5c. These panels show bundling of F-actin into multilayered sheets and display the association between the basal termini of the IMCSFs and basal F-actin in *C. parvum*. **(b)** 2-D slice from a basal end tomogram showing tight bundling of F-actin. The pink box denotes the region that is enlarged in the right panel. The periodicity in the features of F-actin is indicative of the helical arrangement of actin monomers within the filament. **(c)** 2-D slice from a tomogram of a neuron taken from a previously published study[35] showing F-actin bundling. The pink box denotes the region that is enlarged in the right panel. The periodicity in these filaments closely resembles that of *C. parvum* filaments, strongly suggesting that the latter are indeed F-actin. Scale bars are 100 nm in panel a and left panels of b and c, and 10 nm for right panels of b and c.

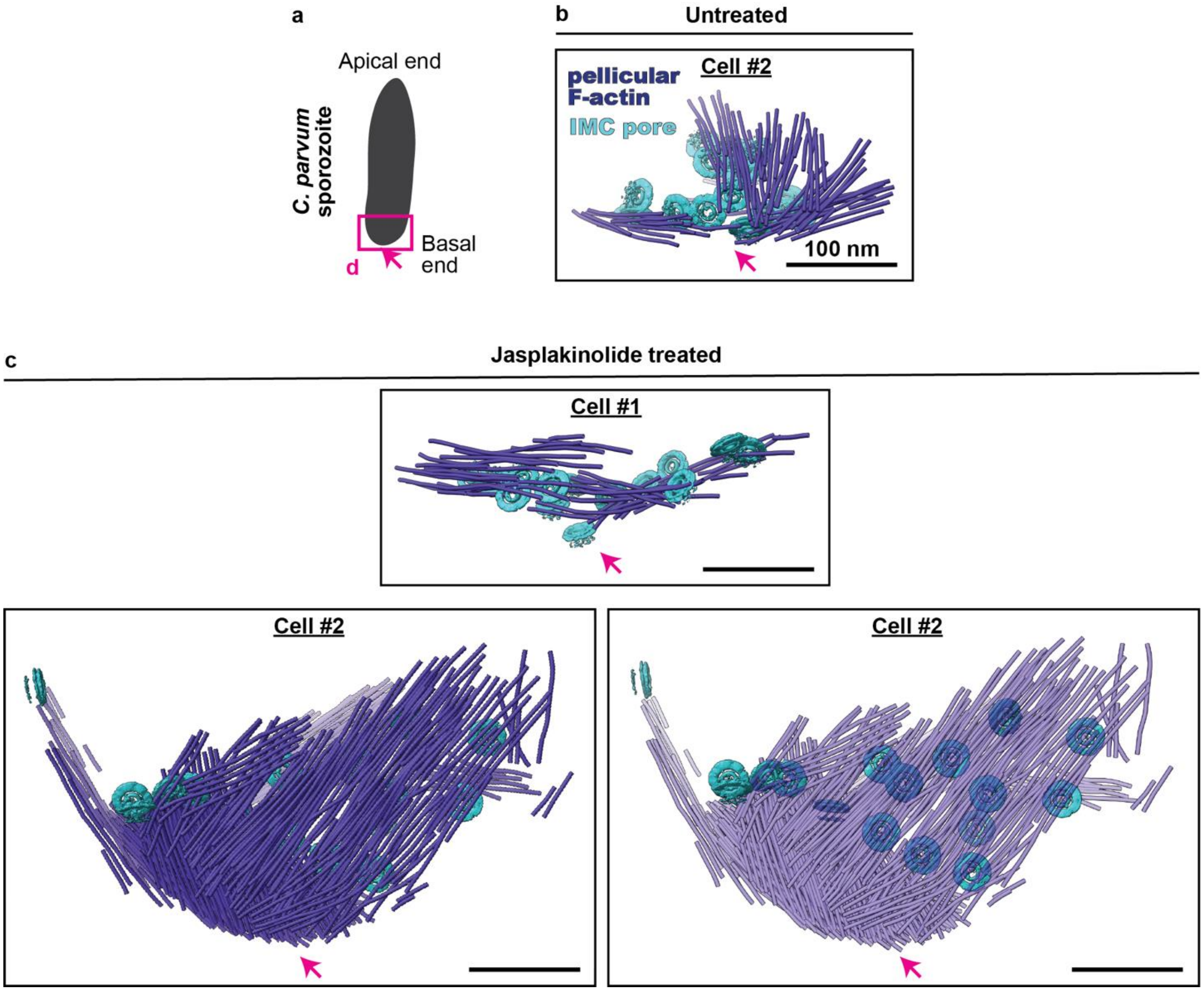

Supplementary Figure 5. Segmentation of F-actin and IMC pores at the basal end of *C. parvum* sporozoites. (a) Schematic of a *C. parvum* sporozoite. The pink box denotes the basal end. **(b)** 3-D segmentation of basal F-actin in another untreated parasite cell (in addition to that shown in Fig. 5d) overlaid with IMC pores. **(c)** 3-D segmentations of basal F-actin in two jasplakinolide-treated parasite cells overlaid with IMC pores. Cell 2 is shown with (right) and without (left) transparency of the F-actin to show the underlying IMC pores. Pink arrows in all panels denote the basal tip. Scale bars are 100 nm.

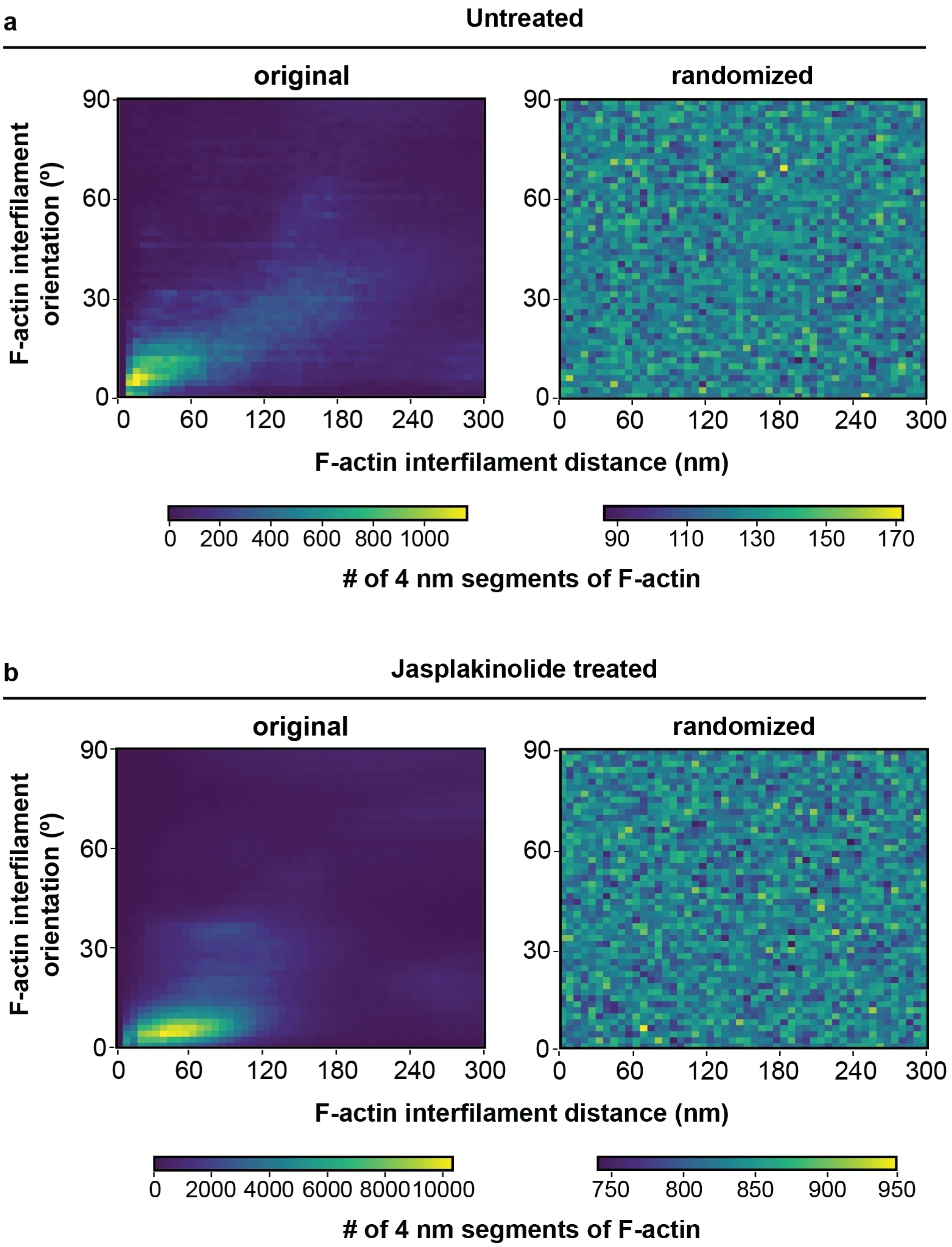

Supplementary Figure 6. F-actin bundling at the basal end of *C. parvum* sporozoites. (a) 2-D histogram of interfilament distance versus orientation of basal F-

actin from 2 untreated *C. parvum* sporozoites (left) along with a randomized control (right)**. (b)** 2-D histogram of interfilament distance versus orientation of basal F-actin from 2 jasplakinolide-treated *C. parvum* sporozoites (left) along with a randomized control (right)**.** These parameters were assessed using 4 nm segments of the filaments.

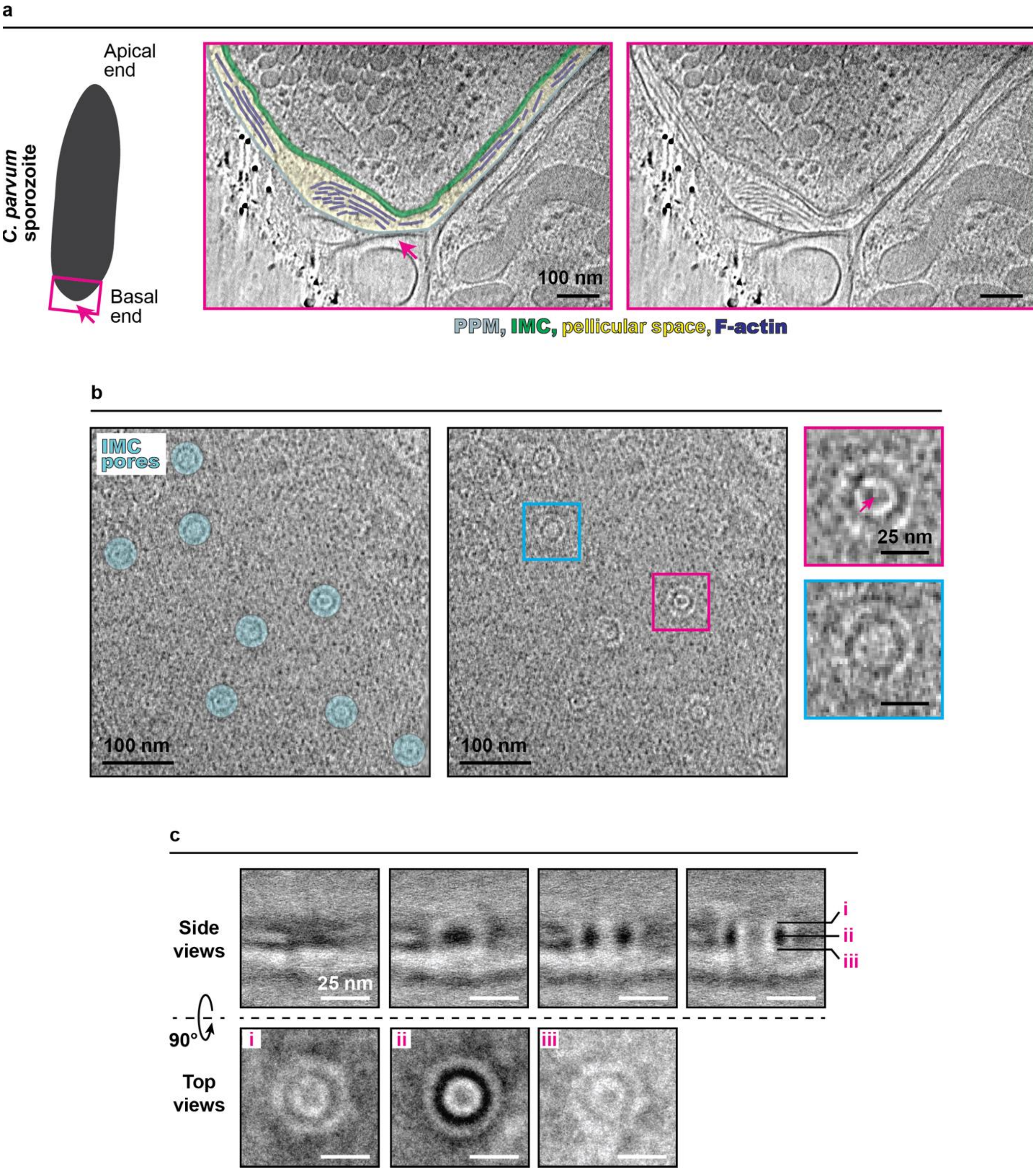

Supplementary Figure 7. F-actin accumulation and IMC pore analyses from basal ends of *C. parvum* sporozoites. **(a)** Schematic of a *C. parvum* sporozoite is shown in the 1st panel. The pink box denotes the basal end. The 2nd and 3rd panels show a 2-D slice through a basal end tomogram with (left) and without (right) color overlay, showing the accumulation of F-actin in the pellicular space causing it to expand. **(b)** 2-D slice

through a basal end tomogram displaying multiple IMC pores with (1st panel) and without (2nd panel) color overlays. The colored boxes denote representative IMC pores and are enlarged on the right. The pink-boxed IMC displays a central density within the pore (pink arrow) that is absent in the cyan-boxed IMC. This density possibly represents cargo passing through the pore. **(c)** 2-D slices from the basal IMC pore subtomogram average, as shown in Fig. 5, but without any applied symmetry. Scale bars are 100 nm in panel a and the larger panels (1st and 2nd) in b, and 25 nm in the smaller panels of b (on the right) and panel c.

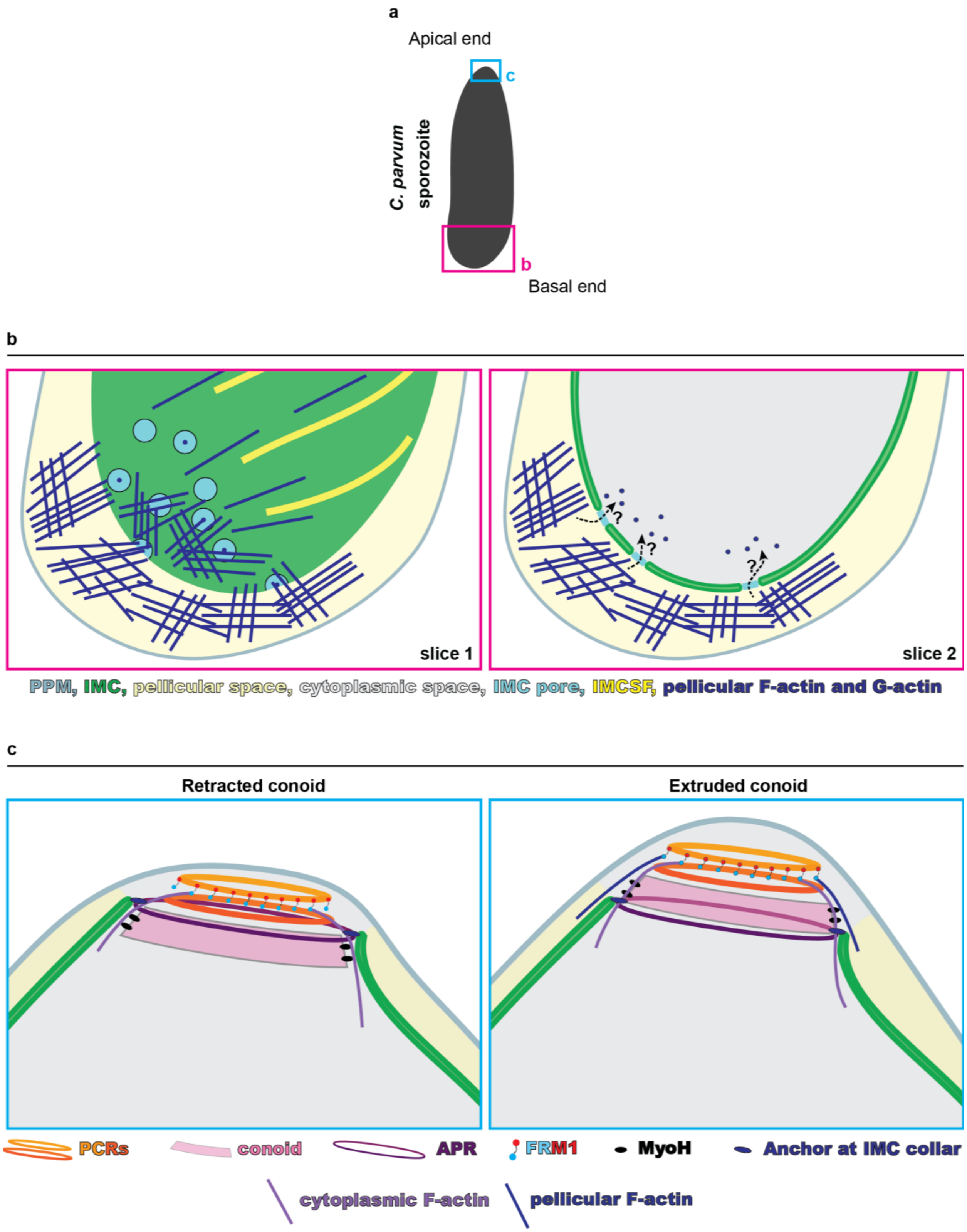

Supplementary Figure 8. Hypothetical models for F-actin recycling at the basal end and coupling of conoid extrusion with pellicular delivery of apically nucleated

F-actin in *C. parvum* sporozoites. **(a)** Schematic of a *C. parvum* sporozoite. Pink box denotes the basal end, shown in panel b and the cyan box denotes the apical end, shown in panel c. **(b)** A hypothetical model for F-actin recycling at the basal end via the IMC pores. On the left (slice 1) is a schematic showing a slice through the basal end at the IMC surface, showing F-actin delivery to the basal cap where they are organized as bundles. On the right is a schematic of a central slice (slice 2) through the basal end, showing the abundant F-actin being depolymerized and transported across the IMC (via the IMC pores) into the cytoplasm in a coupled fashion. **(c)** A speculative mechanistic model for coupling of conoid extrusion with F-actin polymerization and their pellicular delivery. First, when the conoid is retracted (left), FRM1-nucleated F-actin is channeled over the conoid and into the cytoplasm close to the IMC. There, the filaments are potentially anchored to the IMC collar. Using these filaments as "anchored tracks/cables", the conoid-associated MyoH could potentially "pull" the conoid forward to extrude it. Tethering of these filaments at the IMC collar, binding of multiple MyoH molecules (and other as-yet-unknown F-actin binding factors) to each filament, and immobilization of these filaments with respect to the gliding surface through surface adhesins and Glideosome Associated Connector proteins (GAC)[41] could together help to potentially stiffen these actin "tracks". Furthermore, multiple filaments functioning in concert could also help to optimally distribute the force for extrusion. The filaments likely continue to elongate at the PCR in a regulated fashion (or detach from the PCR) until the conoid is fully extruded. Following conoid extrusion, newly nucleated F-actin can now be properly gated into the pellicular space (right).

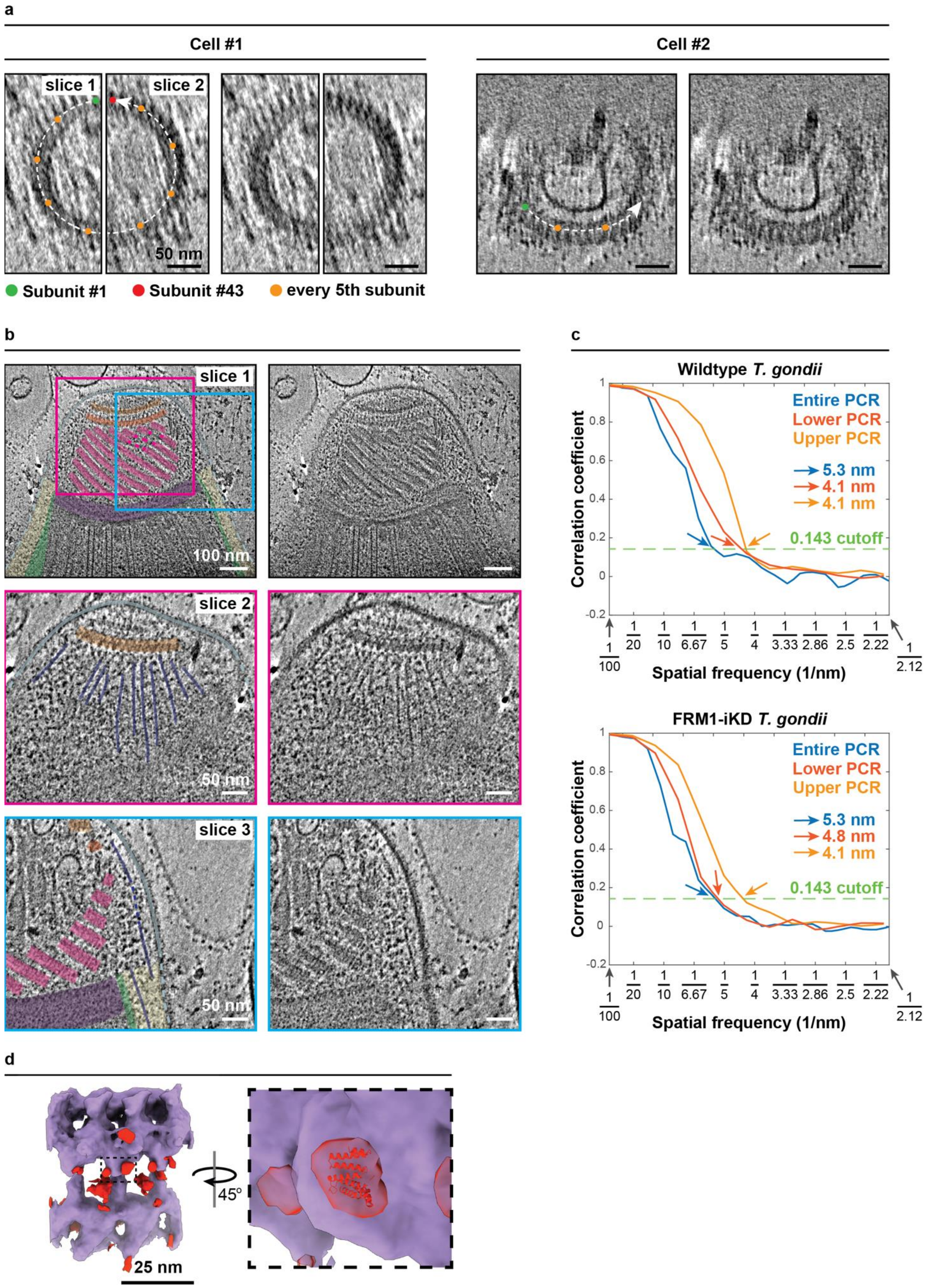
a
Cell #1
Cell #2
slice 1
slice 2
50 nm
Subunit #1
Subunit #43
every 5th subunit
b
slice 1
100 nm
slice 2
50 nm
slice 3
50 nm
c
Wildtype T. gondii
Entire PCR
Lower PCR
Upper PCR
5.3 nm
4.1 nm
4.1 nm
0.143 cutoff
Correlation coefficient
Spatial frequency (1/nm)
FRM1-iKD T. gondii
Entire PCR
Lower PCR
Upper PCR
5.3 nm
4.8 nm
4.1 nm
0.143 cutoff
Correlation coefficient
Spatial frequency (1/nm)
d
45°
25 nm

Supplementary Figure 9. Architecture of *T. gondii* PCRs, and their associated FRM1 and F-actin. (a) 2-D slices through the upper PCR in tomograms of two *T. gondii* tachyzoites, showing a top-down view. For cell #1 on the left, two different slices are used to depict the entire ring since it is slightly deformed, The PCRs in cell #2 on the right are more deformed compared to those in cell #1 but the individual subunits of the upper PCR are better resolved. The 1st panel for each cell is overlaid with colored dots to denote the individual subunits of the PCR. **(b)** 2-D slices from a conoid-extruded *T. gondii* tomogram displaying abundant apical F-actin, with (left) and without (right) color overlays. Color overlays are as follows: light blue for PPM, green for IMC, yellow for the pellicular space, light orange for the upper PCR, dark orange for the lower PCR, pink for conoidal fibers, purple for the APR and dark blue for F-actin. The pink and cyan boxes in the 1st row denote the regions enlarged in the 2nd and 3rd rows, respectively, but they show different Z-slices compared to the 1st row. **(c)** Fourier shell correlation plots for the subtomogram averages of the PCRs from wildtype (top) and FRM1-iKD (bottom) *T. gondii*. **(d)** AlphaFold2 prediction of the TgFRM1 TPR domain structure, fit into one of the missing densities of the FRM1-iKD PCR (in red) at the protruding density (right), zoomed in on the black box in the left panel.

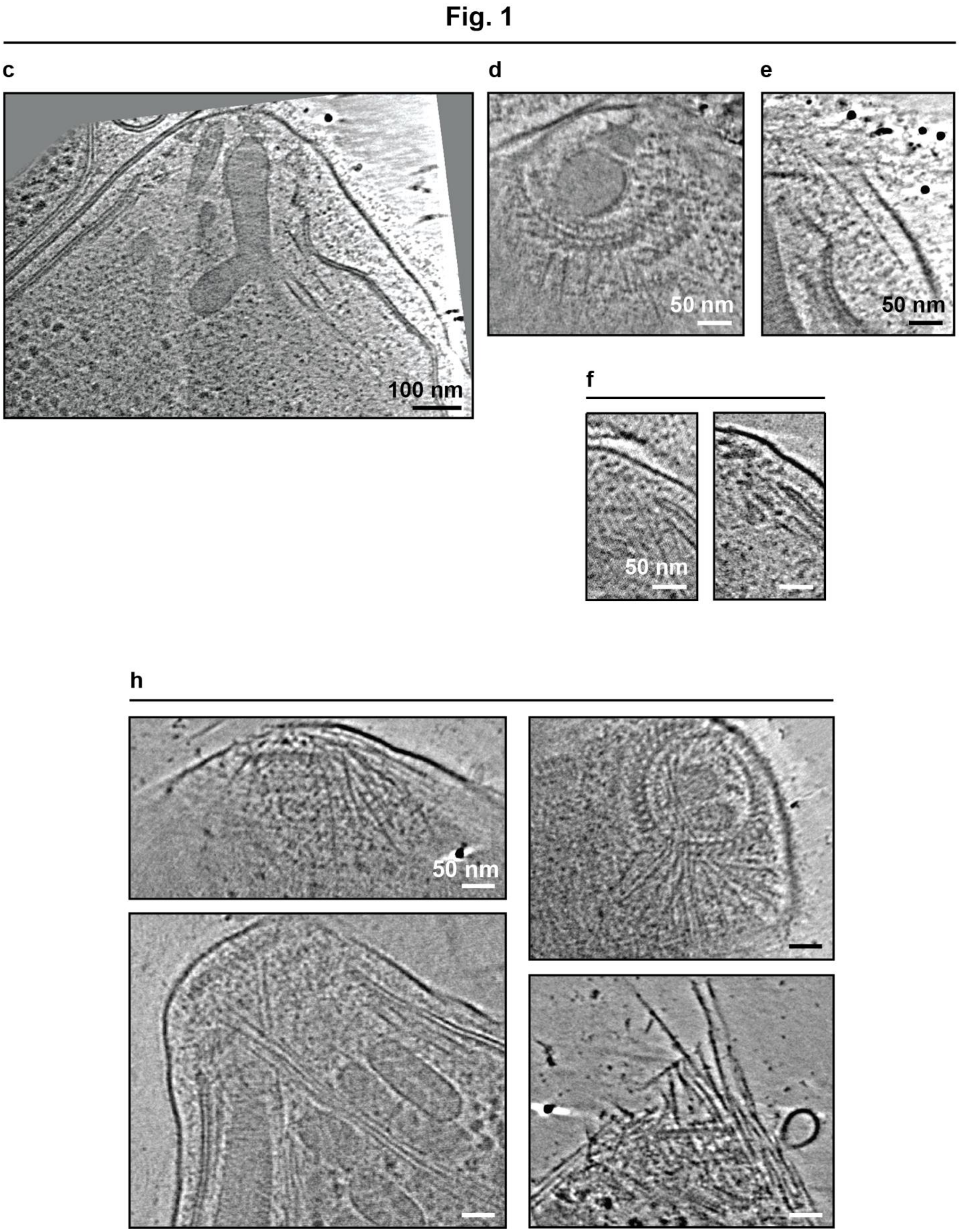

Supplementary Figure 10. **Raw tomogram slices for Fig. 1.** 2-D slices from the apical end tomograms of *C. parvum* shown in Fig. 1 without color overlays.

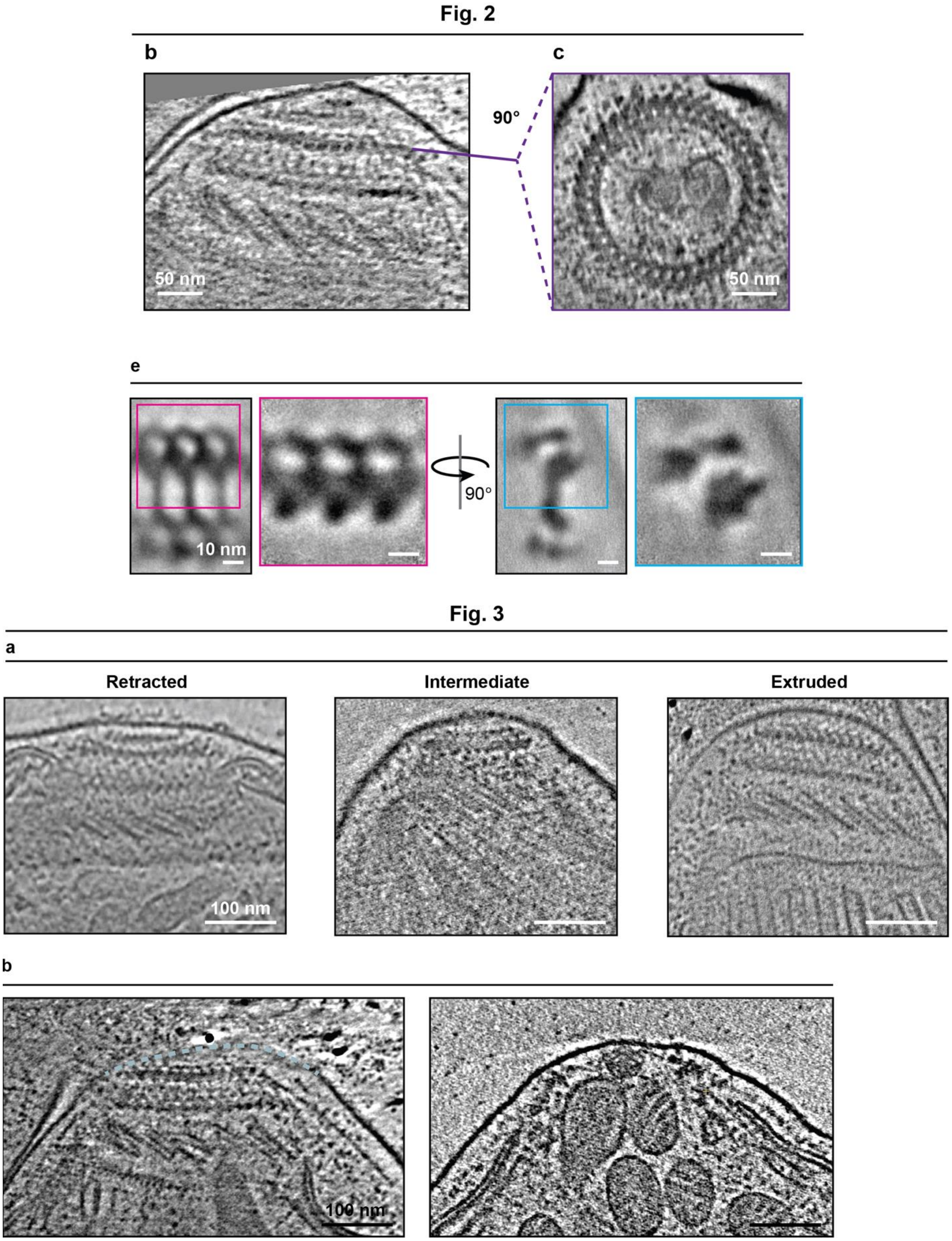

Supplementary Figure 11. **Raw tomogram slices for Figs. 2 and 3.** 2-D slices from the tomograms and subtomogram averages of *C. parvum* shown in Figs. 2 and 3 without color overlays.

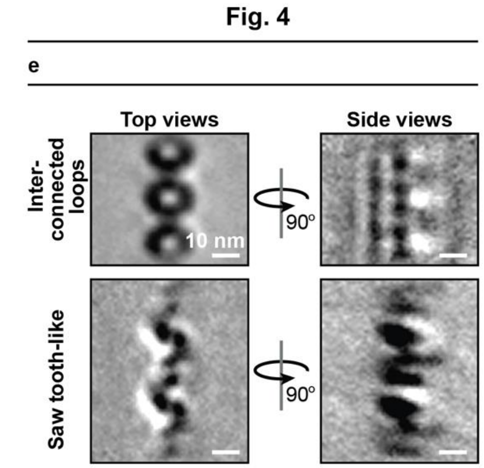

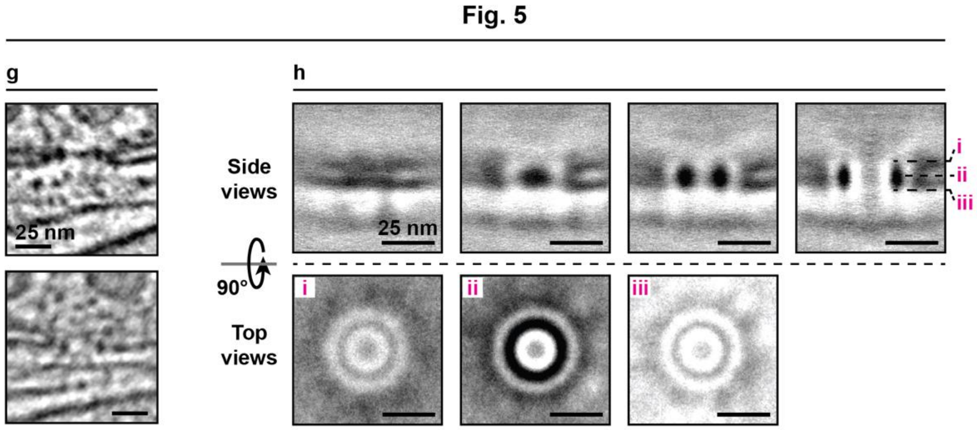

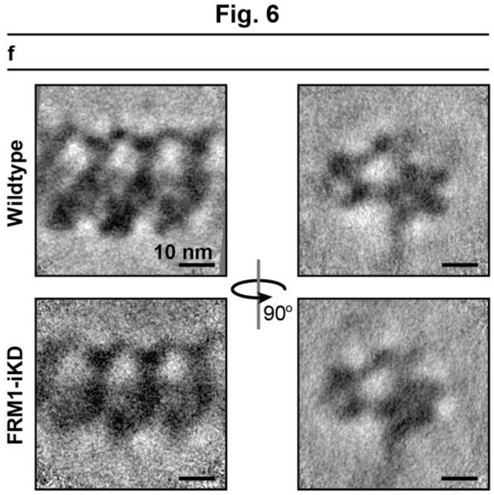

Supplementary Figure 12. **Raw tomogram slices for Figs. 4, 5 and 6.** 2-D slices from the tomograms and subtomogram averages of *C. parvum* shown in Figs. 4, 5, and 6 without color overlays.
