# Supplementary material for: Origin and arrangement of actin filaments for gliding motility in apicomplexan parasites revealed by cryo-electron tomography
Source: Nat Commun. 2023 Aug 9;14:4800. doi: 10.1038/s41467-023-40520-6 (PMC10412601; doi:10.1038/s41467-023-40520-6)
Supplement: Supplementary file 3 — Description of Additional Supplementary Files [file 41467_2023_40520_MOESM3_ESM.pdf]

Description of Additional Supplementary Files

File Name: Supplementary Movie 1
Description: Subtomogram average of the C. parvum PCRs, first showing the front view slices along with the 3-D segmentation of the average, followed by the side view slices (along with the 3-D segmentation of the average again). The movie then shows the refined averages of the upper and lower PCR subunits followed by the annotated structure as shown in Fig. 2.

File Name: Supplementary Movie 2
Description: Subtomogram average of the entire PCR subunit of C. parvum fit back into an apical end tomogram of C. parvum. First, the movie slices through the tomogram to reveal the raw densities, and then reveals the 3-D PCR averages when fit back into the tomogram along with proximal F-actin segmented.

File Name: Supplementary Movie 3
Description: Subtomogram average of the C. parvum IMC surface filaments (IMCSFs). The movie first shows the top view slices through the average (viewing axis and imaging axis perpendicular to the IMC surface) and then reveals the 3-D view of the segmented volume. It then shows side view slices through another average, this time the viewing axis and imaging axis are parallel to the IMC surface such the average readily showed the IMC cross section. Subsequently, when showing the annotated 3-D view of this side view average, the top view average is overlaid in purple to show how the two subtomogram averages align with each other.

File Name: Supplementary Movie 4
Description: Subtomogram average of the C. parvum IMCSF "sawtooth" conformation, first showing the top view slices and the 3-D segmentation of the average, followed by the side view slices along with the 3-D segmentation of the average again.
