# Supplementary material for: Origin and arrangement of actin filaments for gliding motility in apicomplexan parasites revealed by cryo-electron tomography
Source: Nat Commun. 2023 Aug 9;14:4800. doi: 10.1038/s41467-023-40520-6 (PMC10412601; doi:10.1038/s41467-023-40520-6)
Supplement: Supplementary file 8 — Reporting Summary [file 41467_2023_40520_MOESM8_ESM.pdf]

nature portfolio

Corresponding author(s): Yi-Wei Chang

Last updated by author(s): Jul 10, 2023

Reporting Summary

Nature Portfolio wishes to improve the reproducibility of the work that we publish. This form provides structure for consistency and transparency in reporting. For further information on Nature Portfolio policies, see our Editorial Policies and the Editorial Policy Checklist.

Statistics

For all statistical analyses, confirm that the following items are present in the figure legend, table legend, main text, or Methods section.

n/a	Confirmed	
☐	☒	The exact sample size (*n*) for each experimental group/condition, given as a discrete number and unit of measurement
☐	☒	A statement on whether measurements were taken from distinct samples or whether the same sample was measured repeatedly
☐	☒	The statistical test(s) used AND whether they are one- or two-sided<br>*Only common tests should be described solely by name; describe more complex techniques in the Methods section.*
☐	☒	A description of all covariates tested
☐	☒	A description of any assumptions or corrections, such as tests of normality and adjustment for multiple comparisons
☐	☒	A full description of the statistical parameters including central tendency (e.g. means) or other basic estimates (e.g. regression coefficient) AND variation (e.g. standard deviation) or associated estimates of uncertainty (e.g. confidence intervals)
☐	☒	For null hypothesis testing, the test statistic (e.g. *F*, *t*, *r*) with confidence intervals, effect sizes, degrees of freedom and *P* value noted<br>*Give P values as exact values whenever suitable.*
☒	☐	For Bayesian analysis, information on the choice of priors and Markov chain Monte Carlo settings
☒	☐	For hierarchical and complex designs, identification of the appropriate level for tests and full reporting of outcomes
☒	☐	Estimates of effect sizes (e.g. Cohen's *d*, Pearson's *r*), indicating how they were calculated

Our web collection on statistics for biologists contains articles on many of the points above.

Software and code

Policy information about availability of computer code

Data collection	SerialEM 3.8- Tilt series acquisition
Data analysis	IMOD (v4.11.5) – Tomogram reconstruction, segmentation, and modeling; Amira 2019 (ThermoFisher) - Segmentation; UCSF ChimeraX (v1.3) – Segmentation and modeling; MATLAB R2019a and Dynamo (v1.1.509) – Subtomogram averaging; Python 3.7 and 3.8 (Numpy versions 1.19.2 and 1.20.0; Pandas versions 1.1.3 and 1.2.4; MatPlotLib versions 3.3.2 and 3.3.4; Seaborn versions 0.11.0 and 0.11.1) – Analysis and graphing.

For manuscripts utilizing custom algorithms or software that are central to the research but not yet described in published literature, software must be made available to editors and reviewers. We strongly encourage code deposition in a community repository (e.g. GitHub). See the Nature Portfolio guidelines for submitting code & software for further information.

Data

Policy information about availability of data

All manuscripts must include a data availability statement. This statement should provide the following information, where applicable:

- Accession codes, unique identifiers, or web links for publicly available datasets
- A description of any restrictions on data availability
- For clinical datasets or third party data, please ensure that the statement adheres to our policy

Representative tomograms showing an apical and basal end of C. parvum and an apical end of T. gondii are available in the Electron Microscopy Data Bank (EMDB) under accession codes EMD-29754, EMD-29755, and EMD-29753, respectively. Subtomogram averages of the C. parvum PCRs, refined upper PCR, refined lower

PCR are available in the EMDB under the accession codes EMD-29784, EMD-29791, and EMD-29801, respectively. Subtomogram averages of IMCSF top, sawtooth, and side views are available in the EMDB under accession codes EMD-29808, EMD-29809, and EMD-29810, respectively. The subtomogram average of the basal IMC pore is available in the EMDB under the accession code EMD-29835. Subtomogram averages of the wildtype T. gondii PCRs, refined upper PCR, and refined lower PCR are available in the EMDB under accession codes EMD-29832, EMD-29826, and EMD-29827, respectively. Subtomogram averages of the FRM1-iKD T. gondii PCRs, refined upper PCR, and refined lower PCR are available in the EMDB under the accession codes EMD-29838, EMD-29839, and EMD-29840, respectively. Source data are provided with this paper.

Research involving human participants, their data, or biological material

Policy information about studies with human participants or human data. See also policy information about sex, gender (identity/presentation), and sexual orientation and race, ethnicity and racism.

Reporting on sex and gender	N/A
Reporting on race, ethnicity, or other socially relevant groupings	N/A
Population characteristics	N/A
Recruitment	N/A
Ethics oversight	N/A

Note that full information on the approval of the study protocol must also be provided in the manuscript.

Field-specific reporting

Please select the one below that is the best fit for your research. If you are not sure, read the appropriate sections before making your selection.

☒ Life sciences ☐ Behavioural & social sciences ☐ Ecological, evolutionary & environmental sciences

For a reference copy of the document with all sections, see nature.com/documents/nr-reporting-summary-flat.pdf

Life sciences study design

All studies must disclose on these points even when the disclosure is negative.

Sample size	No statistical method was used to predetermine sample size. Instead, sample sizes were arbitrarily chosen based on previous, similar tomographic analyses - Nature Communications, 17 Aug 2021, vol 12, article 4983 (DOI: 10.1038/s41467-021-25309-9)
Data exclusions	No data was excluded from this study.
Replication	228 Cryptosporidium parvum tomograms were collected (119 untreated from three biological replicates, 109 jasplakinolide-treated from two biological replicates). 128 Toxoplasma gondii tomograms were collected (100 wildtype from three biological replicates, 28 Formin1 knockdown from one biological replicate). For each sample, except the T. gondii Formin1 knockdown, multiple frozen grids from multiple sample preparation sessions were imaged over several sessions. For the T. gondii Formin1 knockdown, one frozen grid was imaged. All attempts at replication were successful.
Randomization	Samples (parasites) were allocated randomly to either be drug treated or untreated. Tomograms to be analyzed were first filtered to include those with the best contrast, and then selected at random.
Blinding	No blinding efforts were taken during experiments since experimental design involved specific drug treatments and imaging that are independent of experimenter bias. No blinding efforts were taken during analyses since ground rules were set to eliminate bias.

Reporting for specific materials, systems and methods

We require information from authors about some types of materials, experimental systems and methods used in many studies. Here, indicate whether each material, system or method listed is relevant to your study. If you are not sure if a list item applies to your research, read the appropriate section before selecting a response.

Materials & experimental systems

n/a	Involved in the study
☒	☐ Antibodies
☐	☒ Eukaryotic cell lines
☒	☐ Palaeontology and archaeology
☒	☐ Animals and other organisms
☒	☐ Clinical data
☒	☐ Dual use research of concern
☒	☐ Plants

Methods

n/a	Involved in the study
☒	☐ ChIP-seq
☒	☐ Flow cytometry
☒	☐ MRI-based neuroimaging

Eukaryotic cell lines

Policy information about cell lines and Sex and Gender in Research

Cell line source(s)	Human foreskin fibroblasts (HFF - ATCC, CRL 1634)
Authentication	No additional authentication done by us.
Mycoplasma contamination	HFF cells tested negative for Mycoplasma contamination.
Commonly misidentified lines (See ICLAC register)	None.
